# Factors affecting motivation for receiving a booster dose of the COVID-19 vaccine among Japanese university students and staff: a cross-sectional questionnaire survey

**DOI:** 10.1038/s41598-024-58603-9

**Published:** 2024-04-05

**Authors:** Sho Uchida, Shunsuke Uno, Masahiro Kondo, Yoshifumi Uwamino, Ho Namkoong, Tomoyasu Nishimura, Kana Misawa, Shoko Kashimura, Kei Yamato, Tamami Ishizaka, Kengo Nagashima, Yuko Kitagawa, Naoki Hasegawa

**Affiliations:** 1 Department of Infectious Diseases, Keio University School of Medicine, 35, Shinanomachi, Shinjuku-Ku, Tokyo, 160-8582 Japan; 2 Biostatistics Unit, Clinical and Translational Research Center, Keio University Hospital, 35 Shinanomachi, Shinjuku-Ku, Tokyo, Japan; 3 Graduate School of Health Management, Keio University, 4411 Endo, Fujisawa-Shi, Kanagawa, Japan; 4 Department of Laboratory Medicine, Keio University School of Medicine, 35, Shinanomachi, Shinjuku-Ku, Tokyo, 160-8582 Japan; 5 Keio University Health Center, 2-15-45, Mita, Minato-Ku, Tokyo, 108-0073 Japan; 6 Division of Pharmacodynamics, Keio University Faculty of Pharmacy, 1-5-30 Shibakoen, Minato-Ku, Tokyo, 105-8512 Japan; 7 Department of Surgery, Keio University School of Medicine, 35, Shinanomachi, Shinjuku-Ku, Tokyo, 160-8582 Japan

**Keywords:** Coronavirus disease 2019, Motivation for vaccination, Vaccine promotion, Health belief model, Questionnaire, Adverse events, Disease prevention, Patient education, Viral infection

## Abstract

Understanding the factors that influence people’s decisions regarding vaccination is essential to promote vaccination. We aimed to clarify the motivations for receiving booster vaccines. We conducted a paper-based questionnaire distributed during January–February 2022 involving students and faculty staff who received the first COVID-19 vaccination at the mass vaccination program during June–September 2021 at Keio University. A total of 1725 participants were enrolled, and all completed the survey. Among these, 64.9% reported a significant adverse event (AEs) affecting daily life after the second vaccine. “Fear of severe COVID-19 illness” (72.6%) was the most common reason for getting vaccinated, followed by “concern of infecting others” (68.4%) and “fear of COVID-19 infection itself” (68.3%). Television emerged as the most influential source of information (80%), followed by university information (50.2%) and social networking sites (42.8%). Multivariate analysis revealed “fear of severe COVID-19 illness”, “fear of COVID-19 infection itself”, and “trust in the efficacy and safety of the vaccines in general” were significantly correlated with willingness to receive paid vaccinations. The severity of AEs and source of information were not related to participants’ willingness to receive booster vaccinations. Participants with positive reasons for vaccination were more likely to accept a third dose.

## Introduction

Vaccines against severe acute respiratory syndrome coronavirus 2 (SARS-CoV-2) have been administered worldwide since the end of 2020 with the aim of achieving herd immunity against a pandemic using the first commercially available technology of mRNA vaccines^1^. However, the waning of vaccine efficacy^2–5^ and emergence of variant strains make it challenging to eradicate COVID-19 with vaccines alone; therefore, booster vaccinations are crucial^6^.

Providing adequate and accurate information to vaccine recipients is an important aspect for promoting vaccination^7–10^. Attitudes toward vaccines vary across demographic groups^11–13^; therefore, vaccine promotion strategies need to consider country or region-specific factors that motivate people to receive vaccines. Various factors have been reported to be associated with willingness to receive vaccination in Japan; for example, high levels of knowledge relating to vaccines^14^, information provided on LINE (a kind of social-network service provided by LY Co., Tokyo, Japan) or at workplace/school^15^, and several items in the health belief model (HBM, which is a widely used theory for predicting preventive health-related behavior) have been reported to be associated with the increased uptake of vaccination^14–17^. However, these factors are likely to be confounding factors; comprehensive assessments of the factors are scarce, and motivation for vaccination is still poorly understood, especially in Japan.

The present study aimed to address this gap in the knowledge by clarifying the motivations for receiving COVID-19 booster vaccinations in Japan. To this end, we analyzed the factors contributing to vaccine uptake from different perspectives. The findings presented here provide important information for the promotion of vaccines and future vaccination programs.

## Material and methods

### Participants' recruitment and data collection

This study was conducted through distribution of a paper-based cross-sectional questionnaire survey. The questionnaire (translated into English) is included in the Supplementary Material. From June 21 to September 3, 2021, Keio University's Mita Campus hosted mass vaccinations for the first series of doses (given twice, one month apart). Keio University is a general university located in the center of Tokyo, with a student population of over 30,000. Students and faculty staff who received vaccines through the program were invited to participate in this study via intranet announcements, the university's bulletin board, and Twitter (@keio_covid). Potential participants applied via our website from January to February 2022, the period before the participants received their third dose.

### The questionnaire

The questionnaire included four sections: the first included basic information about the participants; the second included items about adverse events (AEs) after vaccination; the third included items about the history of COVID-19; and the final included items about thoughts about this vaccine and vaccines in general. The final section included questions such as "Would you like to receive the third vaccination?" and "If the third vaccination required charges, how much would you be willing to pay for it?" to further stratify participants' attitudes toward vaccines^18^.

The questionnaire consisted of six A4 pages and took approximately 10 min to complete. Questionnaires were mailed to the study participants in advance, who were asked to fill them out ahead of time. Completed questionnaires were brought to our study site by participants, where they were collected. The data were analyzed anonymously, and items that were not completely filled out were also included in the analysis.

The survey was conducted simultaneously with another study involving the measurement of anti-spike antibody titers from blood samples. Participants received 1000 JPY and the information on their antibody titer in return for their participation. We did not include antibody titers in the analysis.

### Statistics

We performed modified Poisson regressions to estimate the influence of each variable on participants’ willingness to receive the third vaccination, which they had to pay for^19^. Items were scored using a five-point Likert scale and then analyzed by converting into binary variables according to whether they were positive (those ranked 4 or 5) or not positive (those ranked 1–3). The price paid for the third vaccination was analyzed as an ordinal variable, and the influence of each variable was estimated by ordinal logistic regression with a proportional odds model^20^. In ordinal logistic regression, participants who indicated that they would choose not to receive a third vaccination were assigned to the lowest class. Univariate regression analyses were performed using modified Poisson and ordinal logistic regression, followed by multivariate regression analysis using a model that included all variables to control for effects of other variables (e.g., confounding). Risk ratios and 95% confidence intervals (CIs) were estimated in modified Poisson regression, and odds ratios (OR) and 95% CIs were estimated using ordinal logistic regression.

All statistical analyses were performed by available case analysis using R version 4.2.1 (The R Foundation for Statistical Computing; Vienna, Austria).

### Ethical approval and consent to participate

The study has been carried out in accordance with The Code of Ethics of the World Medical Association (Declaration of Helsinki) and was approved by the Keio University School of Medicine Ethics Committee (approval no. 20211089), and written informed consent was obtained from each participant.

## Results

A total of 49,320 people were vaccinated at our university campus during the period of interest, with 1725 (~ 3.5%) of them participating in this study. All enrolled participants completed the questionnaire. The participants' characteristics are summarized in Table 1. Approximately 66.4% of the participants were undergraduate and graduate students, while 33.4% were faculty staff. Fewer than 10% of the participants had underlying medical conditions, with hypertension being the most common (3.1%). There were four pregnant women. All had received two doses of the vaccine with no booster dose. Thirty-six subjects (2.1%) had a history of COVID-19, with seven being asymptomatic, 26 having mild disease, and the remaining three being uncertain. In addition, 10 patients had a long COVID, defined by multisystemic condition comprising often severe symptoms that follow a SARS-CoV-2 infection^21^, six had a taste and smell disorder, and two complained of fatigue.

**Table 1:** Participants’ characteristics.

Variable	Value
Age, mean ± SD (min, max)	30.8 ± 13.6 (18–64)
Gender, n (%)	
Male	759 (44.0)
Female	963 (55.8)
Refused to answer	3 (0.2)
Nationality, n (%)	
Japanese	1658 (96.1)
Others	63 (3.7)
NA	4 (0.2)
Affiliation, n (%)	
Students	931 (54.0)
Graduate students	213 (12.3)
Faculty staff	575 (33.3)
Others	5 (0.3)
NA	1 (0.1)
Underlying medical condition, n (%)	
Cancer	9 (0.5)
Chronic obstructive pulmonary disease	1 (0.1)
Chronic kidney disease	0 (0.0)
Hypertension	54 (3.1)
Diabetes	9 (0.5)
Dyslipidemia	27 (1.5)
Immunodeficiency	0 (0.0)
Immunosuppressive drug use	3 (0.2)
Pregnancy, n (%)	
At the time of vaccination	1 (0.1)
At the time of investigation	3 (0.2)
Vaccination status, n (%)	
Fully vaccinated	1725 (100.0)
Boosted (3rd dose)	0 (0.0)
Past history of COVID-19, n (%)	
Previous history of infection	36 (2.1)
No symptoms	7 (0.4)
Mild	26 (1.5)
Moderate	0 (0.0)
Severe	0 (0.0)
NA	3 (0.2)
Long COVID affected	10 (0.58)

**Figure 1:**
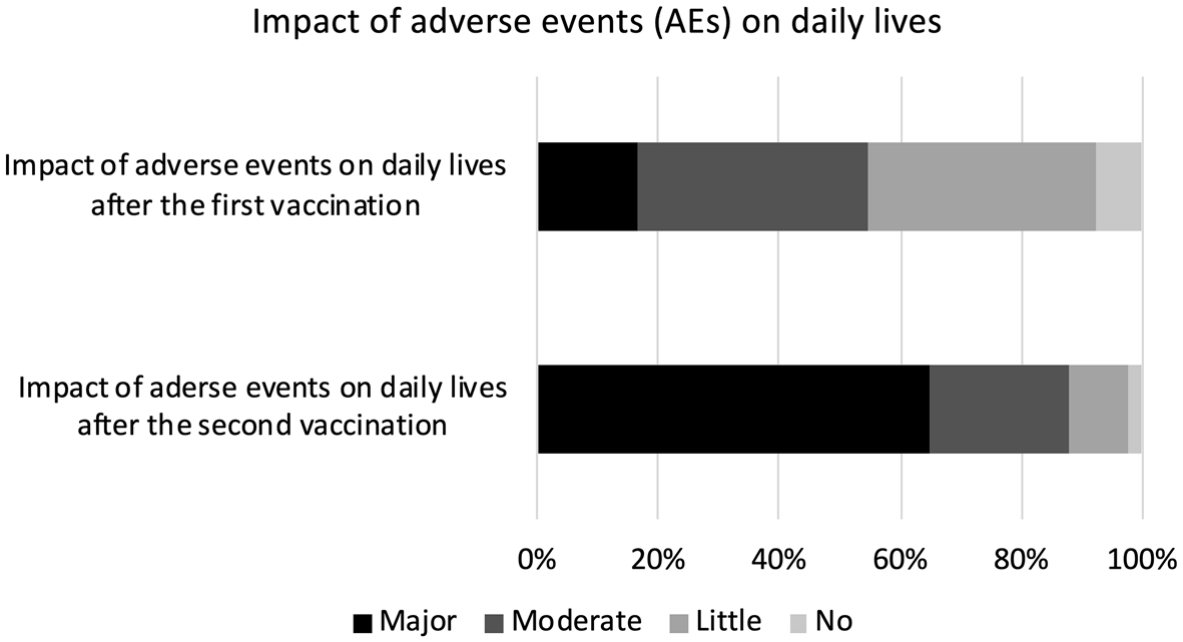
Impact of adverse events (AEs) on daily lives after the first and the second vaccinations. We defined four levels of impact, with major impact indicating that they could not work due to AEs, moderate impact indicating that their work efficacy deteriorated, little impact indicating that their work was little affected, and no impact.

**Figure 2:**
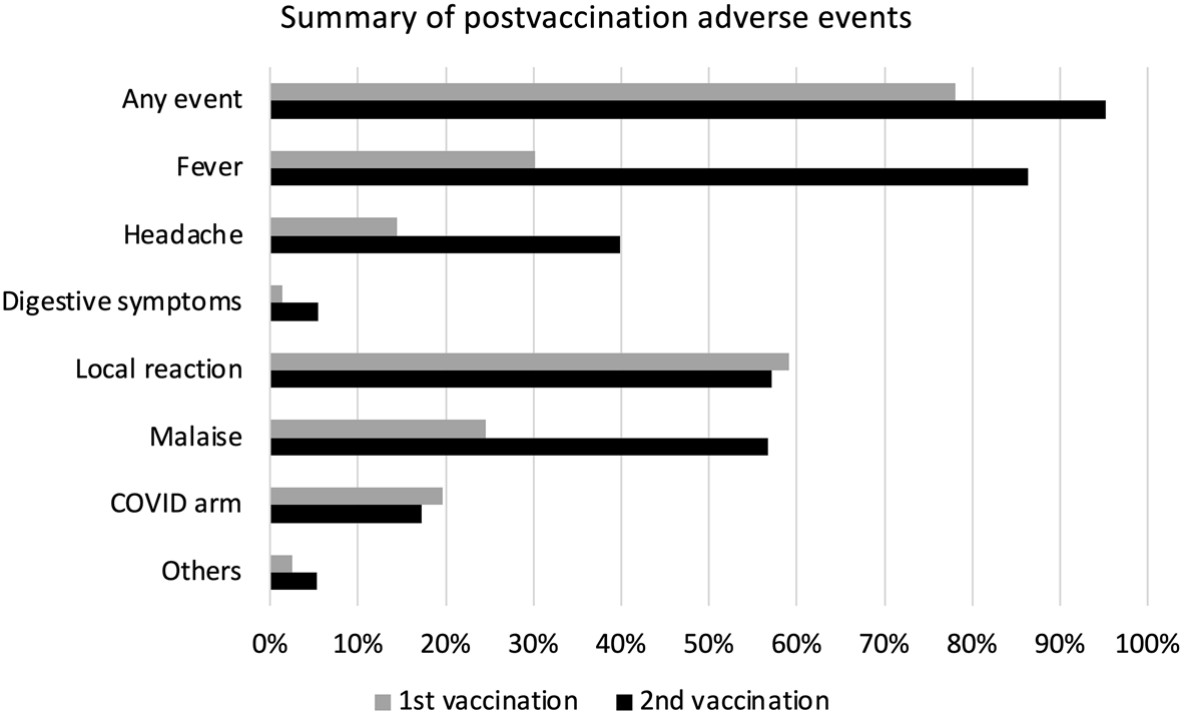
Summary of postvaccination adverse events. We have shown the percentage of participants who experienced each symptom after the first vaccination, as indicated by the gray bar, and after the second, as indicated by the black bar. “COVID arm” is a rash that can appear from a few days to more than a week after vaccination and can sometimes be quite large.

*SD* standard deviation, *NA* not applicable;

Postvaccination AEs are summarized in Figs. 1 and 2. We found that AEs were more common after the second vaccination compared with the first. After the second vaccination, 86.4% reported fever and 64.9% reporting a major impact on daily life (i.e., unable to work due to AEs).

**Figure 3:**
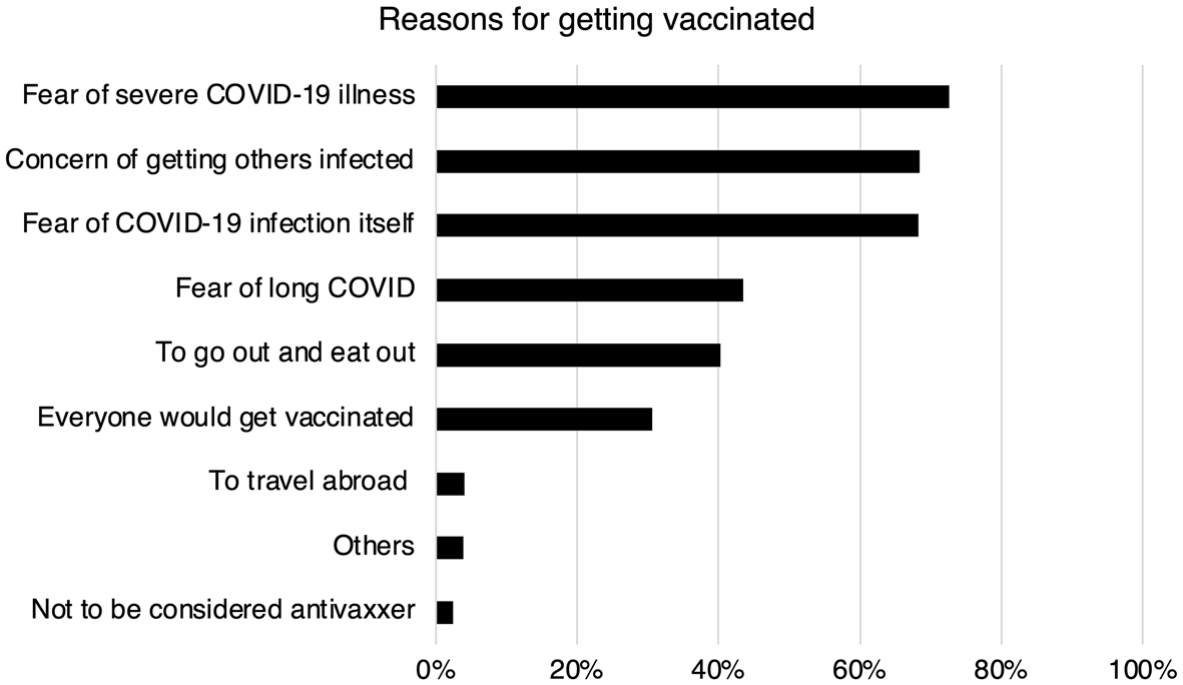
Reasons for getting vaccinated. The percentage of participants who said this was the reason for vaccination is shown for each item.

**Figure 4:**
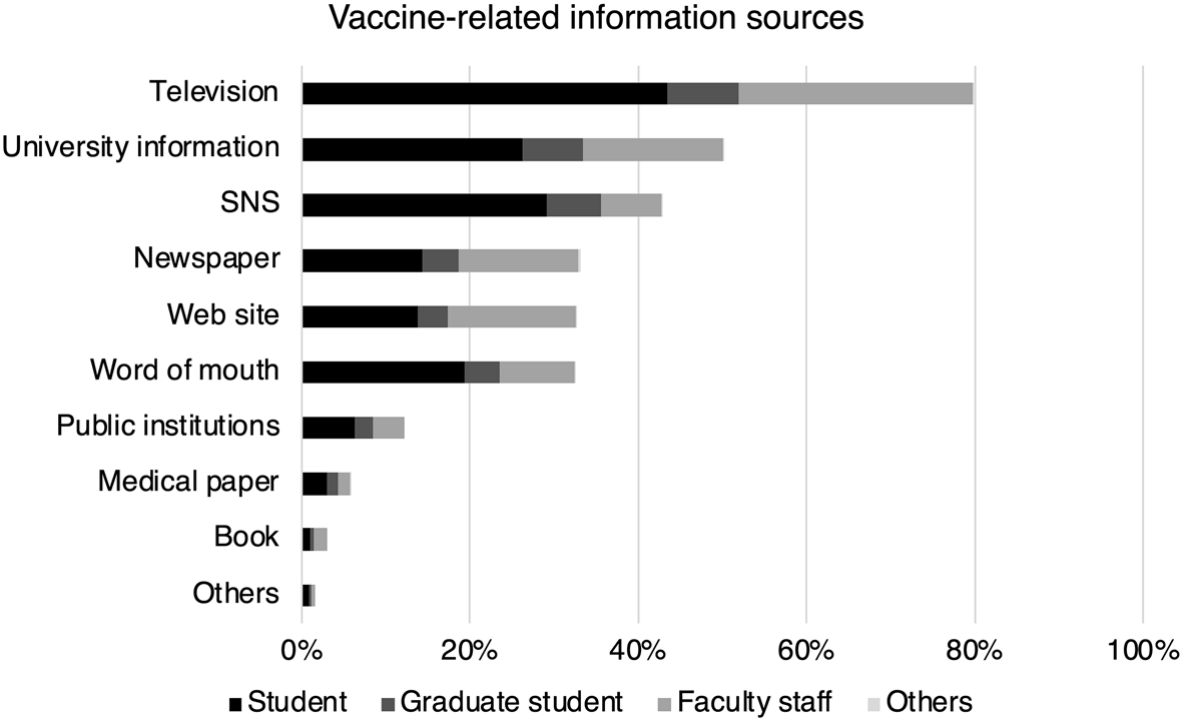
Sources of information on vaccines. We showed where the study participants received their information on vaccination, broken down into affiliations.

The factors that influenced the getting vaccinated, as well as the information resources used, are summarized in Figs. 3 and 4. Fear of severe COVID-19 illness (72.6%) was the most common reason for deciding to vaccinate, followed by concern of getting others infected (68.4%) and fear of COVID-19 infection itself (68.3%). In addition, 30.6% said they would be vaccinated because others around them were. Television was by far the most common information resource, accounting for 80% of respondents, followed by university information (50.2%) and social networking services (42.8%).

**Figure 5:**
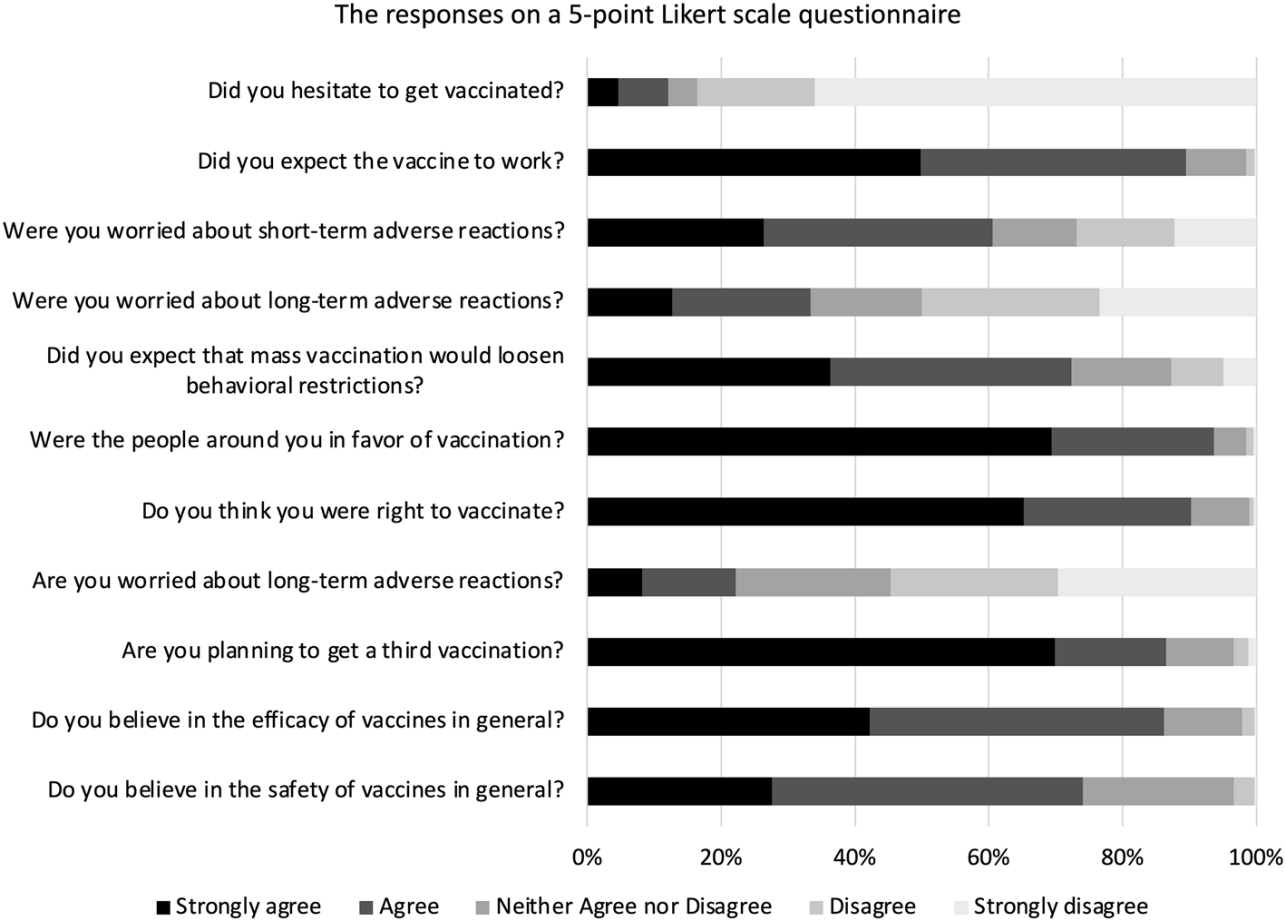
Thoughts on COVID-19 vaccines and vaccines in general. The study participants answered each of the 11 questions on a five-point Likert scale.

The responses on a five-point Likert scale questionnaire are depicted in Fig. 5. Despite some concerns about short- and long-term adverse reactions, 90.3% of respondents did not regret being vaccinated, and 86.5% of responders were optimistic about the third vaccination. Furthermore, 75.2% of respondents were willing to get vaccinated even if they had to pay for the third vaccination, with the median cost being 3000 JPY (Supplemental Fig. 1).

A complete case analysis using data from 1630 (94.5%) participants without missing values was conducted to analyze the factors influencing participants’ motivation for vaccination. As Table 2 shows, these factors influenced participants’ decision, even in cases where the vaccinations were not free. The Supplementary Table shows the results of our analysis of factors influencing participants’ willingness to pay. In multivariate analysis, the risk ratios and odds ratios with 95% CIs that did not include 1.000 are shown in bold, that is, “Everyone would get vaccinated,” “Fear of COVID-19 infection itself,” “Fear of severe COVID-19 illness,” “Do you hesitate to get vaccinated?,” “Do you think you were right to vaccinate?,” “Student,” “Smoking history,” “Newspapers,” “Did you expect that mass vaccination would loosen behavioral restrictions?”, “Do you believe in the efficacy of vaccines in general?”, and “Do you believe in the safety of vaccines in general?”.

**Table 2:** Modified Poisson regression analysis for paid vaccination.

Variables	Univariate analysis			Multivariate analysis		
	RR	95% CI		RR	95% CI	
Background						
Age	1.005	1.003	1.007	1.001	0.998	1.005
Sex (male as a reference)						
Female	1.000	0.946	1.057	1.003	0.950	1.060
Others	0.890	0.399	1.982	1.265	0.963	1.662
Affiliation (faculty staff as a reference)						
Student	0.880	0.831	0.931	0.923	0.831	1.025
Graduate student	0.902	0.824	0.987	0.910	0.821	1.008
Others	0.985	0.635	1.530	0.833	0.513	1.355
Hypertension	1.142	1.018	1.281	0.993	0.872	1.132
Dyslipidemia	1.039	0.847	1.273	0.972	0.782	1.209
Smoking history	1.017	0.857	1.207	0.969	0.832	1.128
Adverse events						
Impact of adverse events on daily lives after the first dose	1.115	0.961	1.294	1.167	0.977	1.394
Impact of adverse events on daily lives after the second dose	0.970	0.944	0.998	0.981	0.953	1.009
History of COVID-19						
History of COVID-19	0.984	0.956	1.014	0.983	0.954	1.013
Sources of information						
Television	0.967	0.905	1.032	0.958	0.899	1.022
Newspapers	1.095	1.037	1.157	1.041	0.986	1.099
Books	1.098	0.961	1.253	1.039	0.919	1.175
Websites	1.078	1.018	1.140	1.030	0.976	1.088
SNS	0.997	0.943	1.054	1.032	0.974	1.094
Public institutions	1.087	1.018	1.162	1.008	0.946	1.074
Medical papers	1.069	0.965	1.185	1.055	0.963	1.155
University information	1.076	1.018	1.136	1.037	0.983	1.094
Word of mouth	0.979	0.922	1.038	0.993	0.937	1.052
Reasons for vaccination						
Everyone would get vaccinated	0.790	0.735	0.848	**0.886**	**0.825**	**0.951**
Not to be considered antivaxxer	0.711	0.534	0.946	0.832	0.644	1.074
Fear of COVID-19 infection itself	1.257	1.172	1.348	**1.125**	**1.050**	**1.204**
Fear of severe COVID-19 illness	1.274	1.182	1.374	**1.115**	**1.033**	**1.203**
Fear of long COVID	1.107	1.049	1.168	0.995	0.941	1.053
Concern of getting others infected	1.182	1.106	1.264	1.045	0.978	1.117
To go out and eat out	1.067	1.011	1.127	1.058	0.996	1.124
To travel abroad (study abroad, business trip, etc.)	1.075	0.955	1.210	1.037	0.928	1.158
Thoughts before vaccination						
Did you hesitate to get vaccinated?	1.466	1.311	1.639	**1.245**	**1.109**	**1.397**
Did you expect the vaccine to work?	1.419	1.238	1.627	1.082	0.949	1.234
Were you worried about short-term adverse reactions?	1.059	0.998	1.123	1.006	0.949	1.066
Were you worried about long-term adverse reactions?	1.068	1.011	1.129	0.979	0.915	1.048
Did you expect that mass vaccination would loosen behavioral restrictions?	1.029	0.967	1.096	0.963	0.904	1.027
Were the people around you in favor of vaccination?	1.270	1.084	1.486	1.113	0.960	1.291
Current thoughts						
Do you think you were right to vaccinate?	1.998	1.650	2.418	**1.708**	**1.407**	**2.073**
Are you worried about long-term adverse reactions?	1.108	1.047	1.172	0.990	0.924	1.060
Do you believe in the efficacy of vaccines in general?	1.258	1.132	1.398	1.012	0.904	1.133
Do you believe in the safety of vaccines in general?	1.197	1.111	1.288	1.087	1.000	1.181

We performed modified Poisson regressions to estimate the influence of each variable on the willingness to receive the third vaccination (the binary variable). Univariate regression analyses were performed, followed by multivariate regression analysis using a model that included all variables to control for the effects of other variables, such as confounding factors. Risk ratios and 95% confidence intervals (CIs) were estimated and significant values were shown in bold.

## Discussion

We conducted questionnaire survey to investigate the motivation to get vaccination. Many subjects did not regret receiving the vaccine and were willing to receive a third dose, although AEs after vaccination appeared to be much stronger than with conventional vaccines of other diseases. In fact, they believed in the vaccine's efficacy and safety and were willing to receive it at their own discretion, possibly due to the successful dissemination of information about the vaccine's efficacy and safety.

A variety of factors can influence the decision to receive vaccinations. These factors can be socioeconomic, psychological, and informational. We found health beliefs to be the major determinants for hesitancy relating to COVID-19 vaccination. The health belief model (HBM) is one of the most widely used models for understanding vaccination behavior against COVID-19^10,17,18,22^. According to the theory, health-related behavior is determined by a combination of several factors including perceived susceptibility, perceived severity, perceived benefits, perceived barriers, cues to action, and self-efficacy^16,22^. The present study evaluated factors that form part of the HBM items (Table 3); however, some items were unclassifiable, suggesting that vaccination motivation cannot be fully explained by the HBM alone. We found that perceived susceptibility, perceived severity, and perceived benefits are all positively correlated with willingness to receive a third COVID-19 vaccination, consistent with previous reports^22^, but items classified into cues to action in this study were negatively correlated. Other studies have used theories of health behavioral science to analyze vaccination motivations^23–25^; however, the present study and a previous report^26^ found that motivations vary depending on the type and source of information, suggesting that a multimodal approach is essential for effective vaccine promotion.

**Table 3:** Correspondence table between health belief model factors and questionnaire items.

Factors	Questionnaire items^†^
Perceived susceptibility	Fear of COVID-19 infection itself
Perceived severity	Fear of severe COVID-19 illness
Perceived benefits	Do you believe in the efficacy of vaccines in general?
	Did you expect the vaccine to work?
Perceived barriers	–
Cues to action	Newspapers
	Everyone would get vaccinated
Self-efficacy	Did you hesitate to get vaccinated?
	Do you think you were right to vaccinate?
Not present items in the health belief model	Affiliation
	Smoking History
	Were the people around you in favor of vaccination?
	Do you believe in the safety of vaccines in general?

^†^Risk ratio and odds ratio in multivariate analysis with 95% confidence intervals that do not cross 1.000.

Belief in the efficacy and safety of the vaccine, as well as belief in the justification for vaccination, were highly correlated with intention to receive a third vaccination. Positive reasons for vaccination included trusting in the rightness of vaccination and believing in the efficacy and safety of vaccines in general. Participants with passive reasons for vaccination, such as “everyone will get vaccinated”, were more hesitant to vaccinate a third time. Trust is an important factor for vaccine promotion and has been reported to be a denominator for vaccine acceptance^27^. Our results suggest that vaccine promotion in Japan has been somewhat successful; however, our study design may have introduced some bias, as people who are in favor of vaccines may be more likely to participate. We also asked study participants to address those bias about their willingness to pay for vaccination to stratify willingness to vaccinate, based on a previous study^18^. We found similarities between the items involved in participant’s decision to receive a third vaccination. The primary reason for hesitance toward paid-for vaccinations in the students was considered to be financial insecurity. A history of smoking was correlated with a willingness to vaccinate, even if the cost was higher. This may be because of the perceived higher risk of serious illness.

Television was by far the most common source of information about vaccination. The majority of participants were young; although the media landscape is diversifying in Japan, television may remain an important source of information. Previous studies have shown both positive and negative effects of social networking services on vaccination^28^, but our study found neither. Further research is needed to determine how vaccine-related information sources influence vaccination intentions.

This study has some limitations that should be acknowledged. First, the number of participants was small compared with the total number vaccinated in the mass vaccination program. Participants were recruited via intranet announcements, campus bulletin boards, and Twitter; however, it was not possible to contact all 49,320 people who were vaccinated in the mass vaccination. In addition, the study period fell during a university holiday, resulting in low participation. Therefore, the study may have selectively attracted more people who were interested in the vaccine and were willing to be vaccinated, leading to potential bias. However, the percentage of people who reported getting vaccinated for the passive reason of “everyone will get vaccinated” was not significantly different from previous reports^29–31^, suggesting that the selection bias may not be significant.

Second, this study does not clarify the best approach to recruit participants who did not receive vaccination, because all subjects in this study favored vaccination. It is necessary, therefore, to conduct a study in which subjects are randomly selected from a population containing various people with different views on getting vaccinated and asked to complete a questionnaire.

It is also worth noting that the above vaccination arguments presume that the vaccine is effective and safe. Amid a pandemic, promoting the vaccination of the entire population with a vaccine with a completely new mechanism may pose a significant risk. For instance, a study found that the North American university COVID-19 vaccine was likely to cause net-expected harm to young healthy adults—they estimated approximately 18.5 severe AEs for each hospitalization averted, including myocarditis, and 1430–4626 disruptions of daily activities—that were not outweighed by a proportionate public health benefit^32^. The survey results indicate that the promotion in Japan was successful, but the correlation between the high vaccination rate and socioeconomic or public impact must be investigated further.

## Conclusions

The severity of AEs and the source of information are not significantly related to individuals’ willingness to receive booster vaccinations for COVID-19. Factors that are most associated with willingness to receive a third dose are fear of severe COVID-19 illness, fear of COVID-19 infection itself, belief in the rightness of getting vaccination, and trust in the efficacy and safety of the vaccines in general.

### Supplementary Information

Supplementary Information.

## Data Availability

The datasets used and analyzed during the current study are available from the corresponding author upon reasonable request.

## Abbreviations

AEs: Adverse events
CI: Confidence intervals
COVID-19: Coronavirus disease 2019
HBM: Health belief model
SARS-CoV-2: Severe acute respiratory syndrome coronavirus-2
