# Supplementary material for: Factors affecting motivation for receiving a booster dose of the COVID-19 vaccine among Japanese university students and staff: a cross-sectional questionnaire survey
Source: Sci Rep. 2024 Apr 5;14:8009. doi: 10.1038/s41598-024-58603-9 (PMC10997627; doi:10.1038/s41598-024-58603-9)
Supplement: Supplementary file 1 — Supplementary Information. [file 41598_2024_58603_MOESM1_ESM.docx]

**Supplementary figure.** Willingness to pay for the third vaccine. Participants responded by choosing the amount they would be willing to pay for the third vaccination from a range of less than 1,000 JPY to more than 10,000 JPY, separated by 1,000 JPY.

**
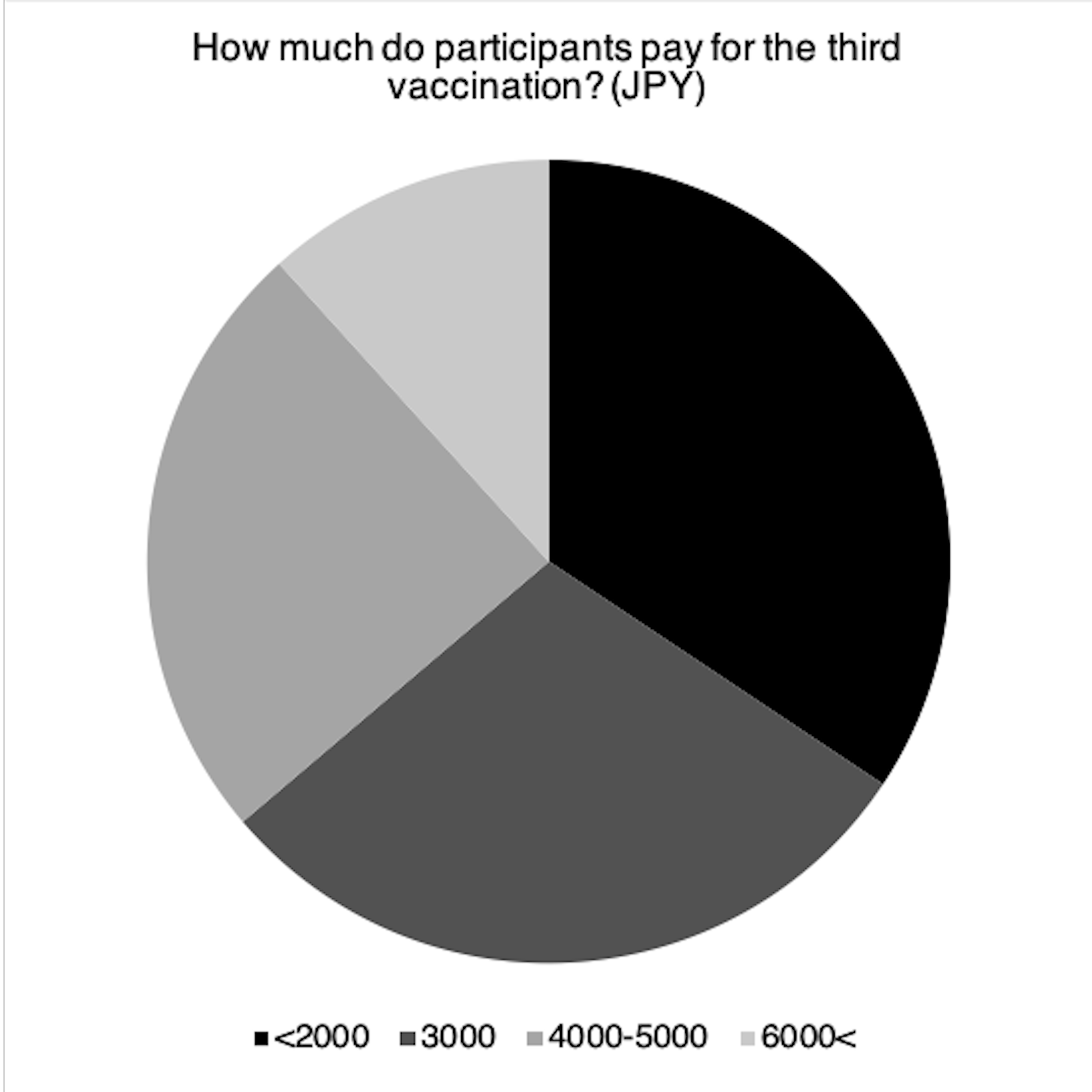
**

| **Supplementary table**. Ordinal logistic regression analysis for vaccination fee. | | | | | | | | |
| --- | --- | --- | --- | --- | --- | --- | --- | --- |
| Variables | | | Univariate analysis | | | Multivariate analysis | | |
|  |  |  | OR | 95% CI | | OR | 95% CI | |
| Background | Age |  | 1.004 | 0.991 | 1.018 | 1.004 | 0.991 | 1.018 |
|  | Sex (male as a reference) | Female | 1.007 | 0.832 | 1.221 | 1.007 | 0.832 | 1.221 |
|  |  | Others | 2.939 | 0.319 | 27.107 | 2.939 | 0.319 | 27.107 |
|  | Affiliation  (Faculty staff as a reference) | Student | 0.664 | 0.447 | 0.987 | **0.664** | **0.447** | **0.987** |
|  |  | Graduate student | 0.775 | 0.521 | 1.151 | 0.775 | 0.521 | 1.151 |
|  |  | Others | 0.300 | 0.053 | 1.590 | 0.300 | 0.053 | 1.590 |
|  | Hypertension |  | 1.289 | 0.740 | 2.245 | 1.289 | 0.740 | 2.245 |
|  | Dyslipidemia |  | 0.788 | 0.365 | 1.673 | 0.788 | 0.365 | 1.673 |
|  | Smoking History |  | 1.994 | 1.062 | 3.738 | **1.994** | **1.062** | **3.738** |
| Adverse events | Impact of adverse events on daily lives after the first dose |  | 1.544 | 0.812 | 2.939 | 1.544 | 0.812 | 2.939 |
|  | Impact of adverse events on daily lives after the second dose |  | 0.943 | 0.858 | 1.036 | 0.943 | 0.858 | 1.036 |
| History  of COVID-19 | |  | 0.966 | 0.869 | 1.073 | 0.966 | 0.869 | 1.073 |
| Sources  of information | Television |  | 0.887 | 0.705 | 1.115 | 0.887 | 0.705 | 1.115 |
|  | Newspapers |  | 1.217 | 1.002 | 1.478 | **1.217** | **1.002** | **1.478** |
|  | Books |  | 1.259 | 0.749 | 2.111 | 1.259 | 0.749 | 2.111 |
|  | Websites |  | 0.976 | 0.801 | 1.189 | 0.976 | 0.801 | 1.189 |
|  | SNS |  | 1.029 | 0.848 | 1.249 | 1.029 | 0.848 | 1.249 |
|  | Public institutions |  | 1.258 | 0.981 | 1.614 | 1.258 | 0.981 | 1.614 |
|  | Medical papers |  | 1.237 | 0.832 | 1.839 | 1.237 | 0.832 | 1.839 |
|  | University information |  | 1.118 | 0.930 | 1.343 | 1.118 | 0.930 | 1.343 |
|  | Word of mouth |  | 1.018 | 0.835 | 1.241 | 1.018 | 0.835 | 1.241 |
| Reasons  for vaccination | Everyone would get vaccinated |  | 0.750 | 0.608 | 0.926 | **0.750** | **0.608** | **0.926** |
|  | Not to be considered antivaxxer |  | 0.563 | 0.294 | 1.060 | 0.563 | 0.294 | 1.060 |
|  | Fear of COVID-19 infection itself |  | 1.270 | 1.029 | 1.567 | **1.270** | **1.029** | **1.567** |
|  | Fear of severe COVID-19 illness |  | 1.240 | 0.989 | 1.554 | 1.240 | 0.989 | 1.554 |
|  | Fear of long COVID |  | 1.119 | 0.919 | 1.363 | 1.119 | 0.919 | 1.363 |
|  | Concern of getting others infected |  | 1.065 | 0.866 | 1.310 | 1.065 | 0.866 | 1.310 |
|  | To go out and eat out |  | 1.137 | 0.929 | 1.391 | 1.137 | 0.929 | 1.391 |
|  | To travel abroad  (study abroad, business trip, etc.) |  | 1.098 | 0.703 | 1.714 | 1.098 | 0.703 | 1.714 |
| Thoughts  before vaccination | Did you hesitate to get vaccinated? |  | 1.628 | 1.227 | 2.163 | **1.628** | **1.227** | **2.163** |
|  | Did you expect the vaccine to work? |  | 1.439 | 1.040 | 1.996 | **1.439** | **1.040** | **1.996** |
|  | Were you worried about short-term adverse reactions? |  | 1.195 | 0.968 | 1.476 | 1.195 | 0.968 | 1.476 |
|  | Were you worried about long-term adverse reactions? |  | 1.083 | 0.866 | 1.354 | 1.083 | 0.866 | 1.354 |
|  | Did you expect that mass vaccination would loosen behavioral restrictions? |  | 0.806 | 0.649 | 1.001 | 0.806 | 0.649 | 1.001 |
|  | Were the people around you in favor of vaccination? |  | 1.594 | 1.074 | 2.379 | **1.594** | **1.074** | **2.379** |
| Current thoughts | Do you think you were right to vaccinate? |  | 2.691 | 1.878 | 3.885 | **2.691** | **1.878** | **3.885** |
|  | Are you worried about long-term adverse reactions? |  | 1.228 | 0.982 | 1.537 | 1.228 | 0.982 | 1.537 |
|  | Do you believe in the efficacy of vaccines in general? |  | 1.263 | 0.927 | 1.721 | 1.263 | 0.927 | 1.721 |
|  | Do you believe in the safety of vaccines in general? |  | 1.438 | 1.127 | 1.836 | **1.438** | **1.127** | **1.836** |
| The amount paid for the third vaccination was analyzed as an ordinal variable, and the influence of each variable was estimated by ordinal logistic regression with proportional odds model. Participants who indicated that they would not receive a third vaccination were assigned to the lowest class. Univariate regression analyses were performed, followed by multivariate regression analysis with a model that included all variables for controlling the effects of other variables, such as confounding. Odds ratios (OR) and 95% confidence intervals (CIs) were estimated. | | | | | | | | |

**Supplementary material.** Questionnaire for a study on the effectiveness of workplace vaccination with COVID-19 vaccine


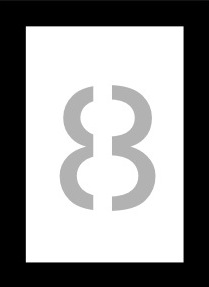

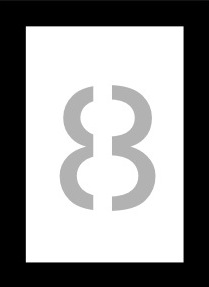

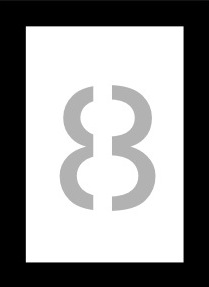

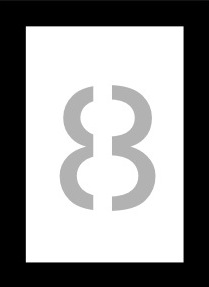

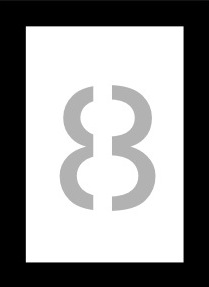


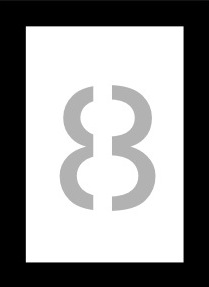

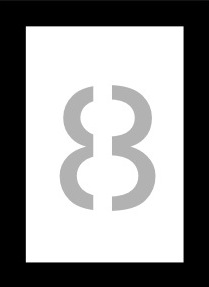

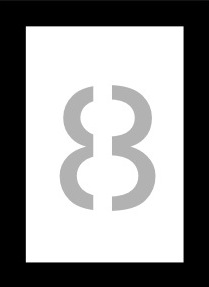
Student/Faculty/Staff ID number（　　　　　　　　　　　　）　　Name（　　　　　　　　　　）

＊Above columns are disconnected after anonymization＊


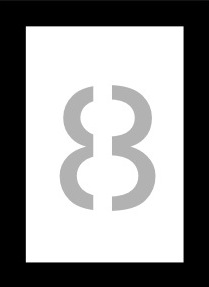

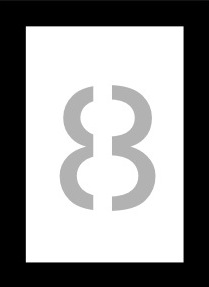

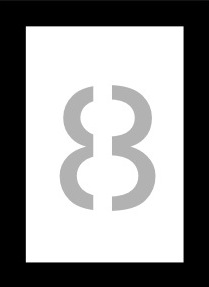

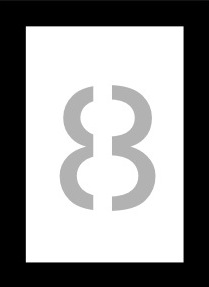


Research ID（filled in by researcher）：　　　　　　　　Date (MM/DD)　　　　　 /

Thank you for your cooperation in the " study on the effectiveness of workplace vaccination with COVID-19 vaccine". We would appreciate your cooperation in answering the following questionnaire regarding information needed for the study.

**①Basic information**


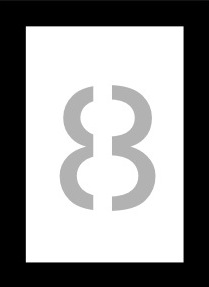

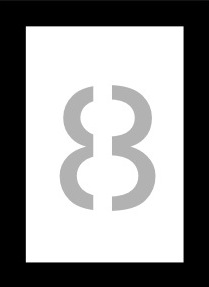
Age：（　　　）years old

**
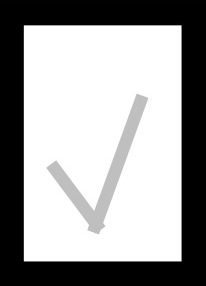

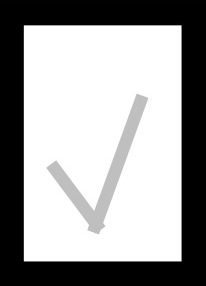

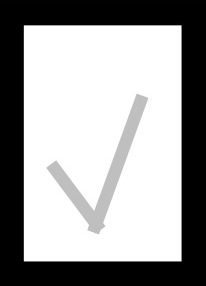
**Sex：（　 Male　 Female No answer）

**
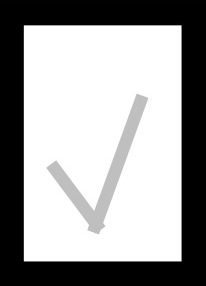

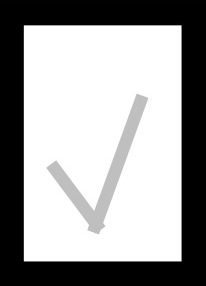
**Nationality：（　Japan　 Others）

**
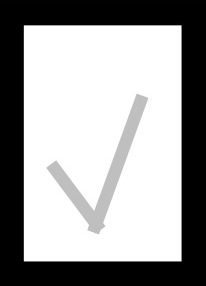

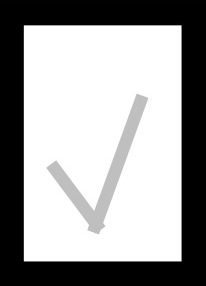

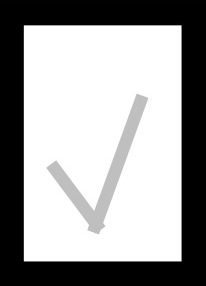

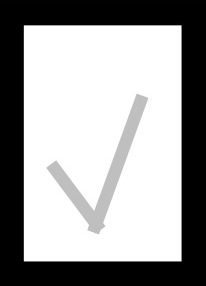
**Affiliation：（　Student　 Graduate student　 Staff/Faculty　 Others）

**
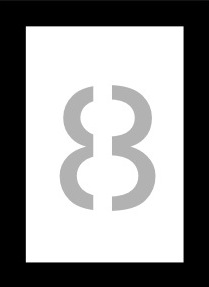

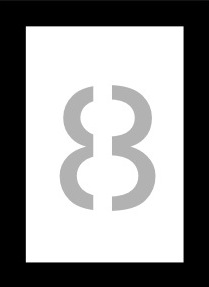

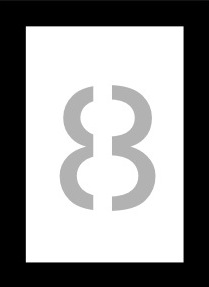
**Height：（　　　 ）cm


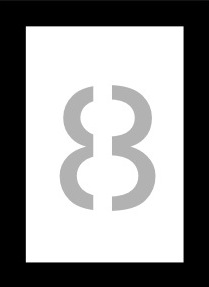

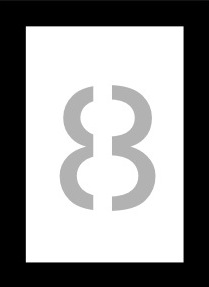
Weight：（　　　）kg

**
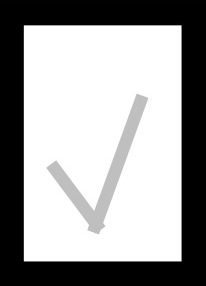

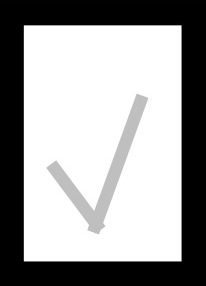

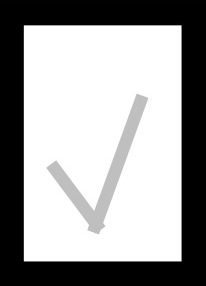

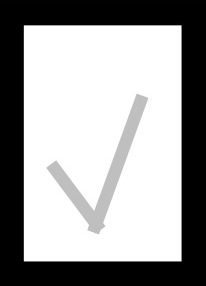
**Underlying diseases： Cancer　 COPD/Emphysema　 CKD　 HT

**
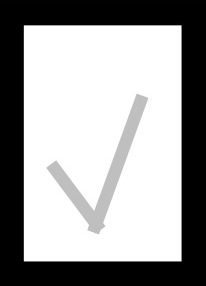

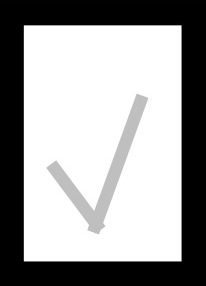

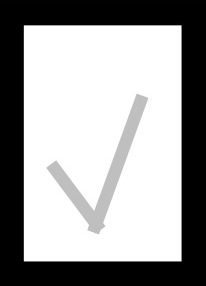
**DM　 DL Smoking history


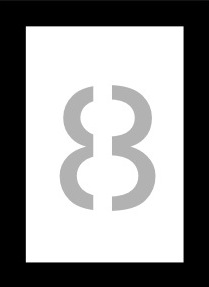

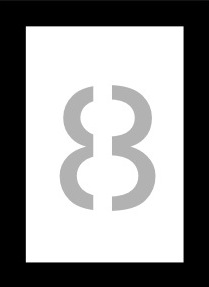

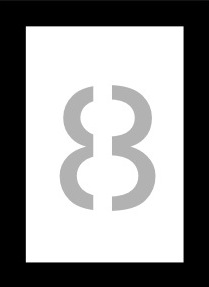

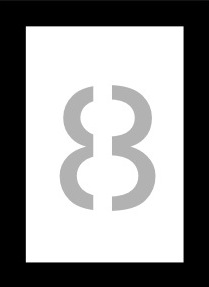
**
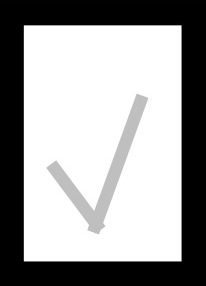
**Immunodeficiency（Details：　　　　　　　 　）

**
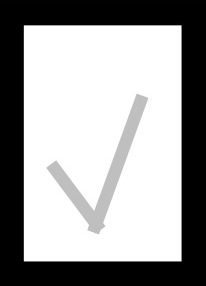
**History of organ transplantation（Year： , Organ： ）

**
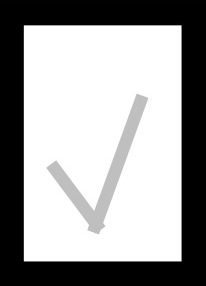
**Use of immunosuppressive drug（Details：　 　 　　　）

**
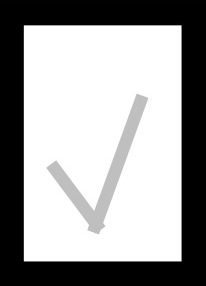
**Use of anticancer drug（Details：　　　　　　　　 　　　 　 ）

**
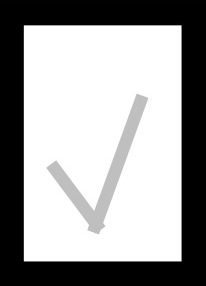
**
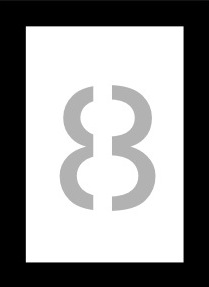

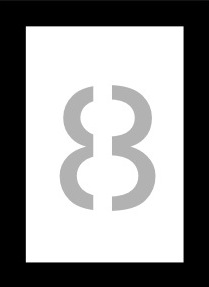
Pregnancy：　Pregnant at the time of vaccination（ weeks）


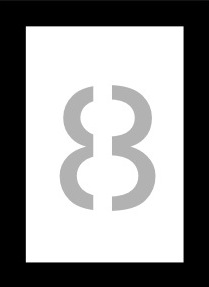

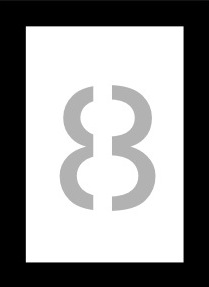
**
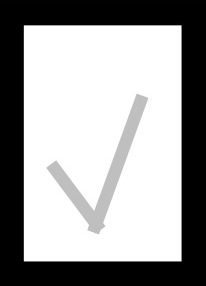
**Currently pregnant（ weeks）

**
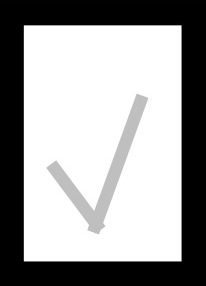

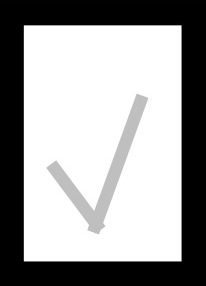

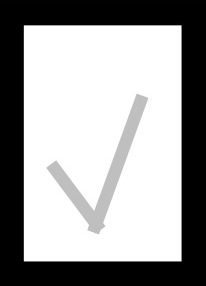

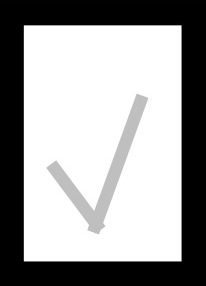

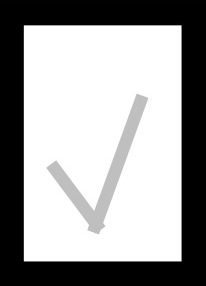
**Allergy： Pollinosis　 Food　 Drug　 Metal　 Atopic dermatitis

**
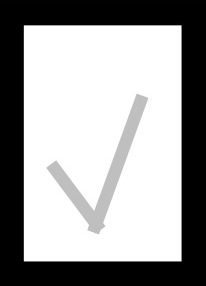

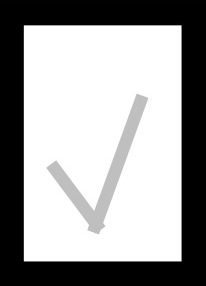
**Anaphylaxis　 Others (Details :　　　　 　 )


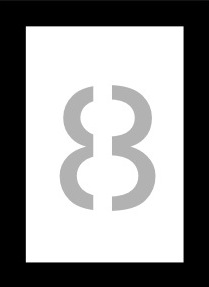

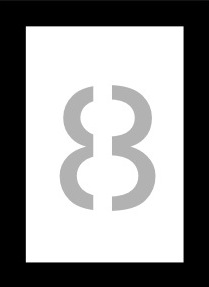

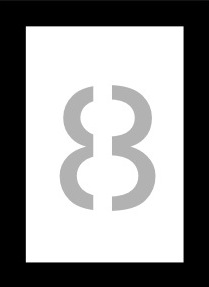

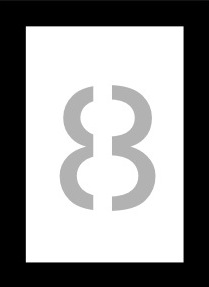
Vaccination date (MM/DD) : 1st dose（ / ）


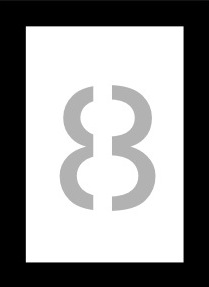

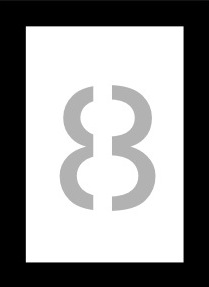

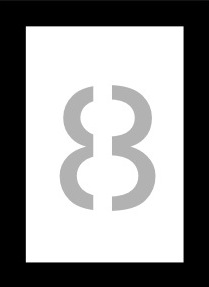

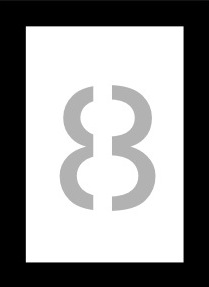
2nd dose（　　　/ 　　）


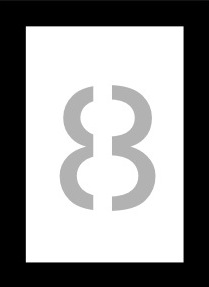

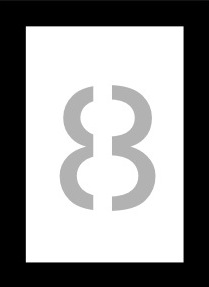
**
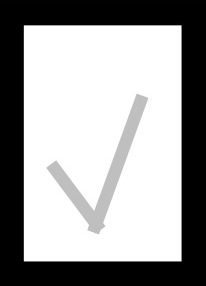
**3rd dose　　Yes


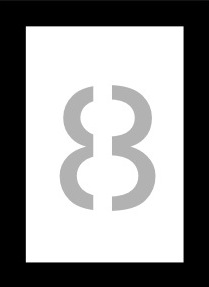

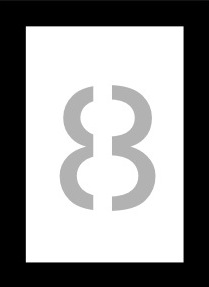
Date（　　　/ 　　 ）

**
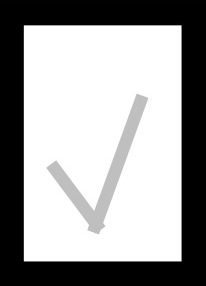

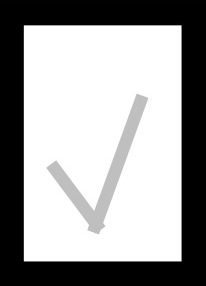
**Type　 BNT162b2　 mRNA-1273

**
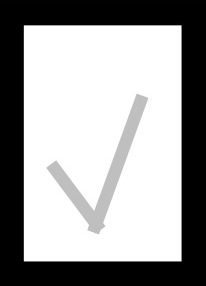
**No

**②Adverse events**

**A. 【After the first dose】Have any physical symptoms appeared?**

**
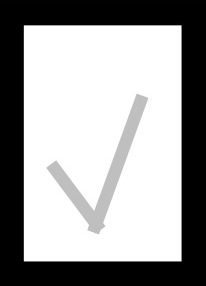

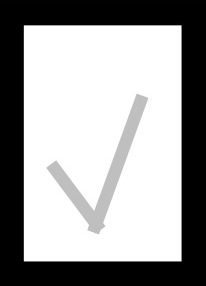
**　 No　　　 Yes

**A-1.** **If you answered "Yes", please mark the symptoms that apply to you.**

**
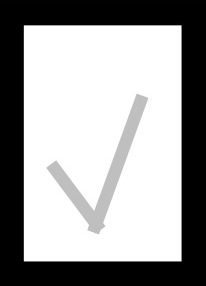

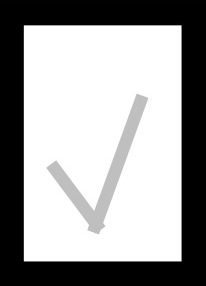
**
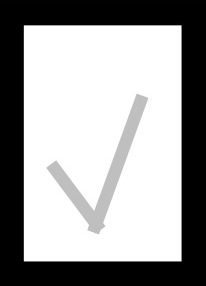
 Fever　Headache GI symptoms（nausea, vomiting, diarrhea）


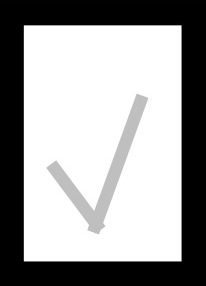

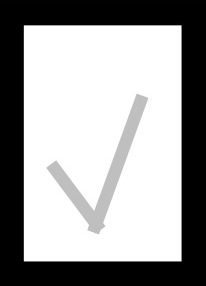
　Local reactions（pain, swelling, redness, itch）　Malaise


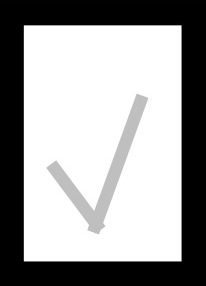
　　COVID arm


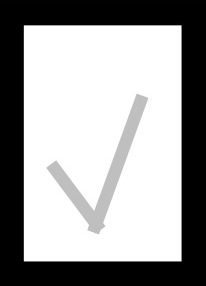
　Others（Details: 　　　　　　　　　　　　　　　　　　　　）

**A-2. To what extent have your physical symptoms affected your life?**

**
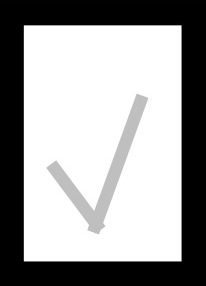
**　　　Major impact（ex. Couldn’t work due to AE）


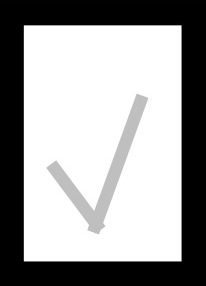
Moderate impact（ex. Work efficiency deteriorated.）


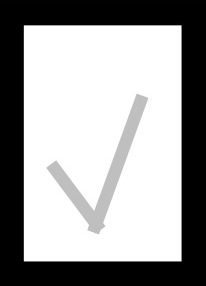
Little impact


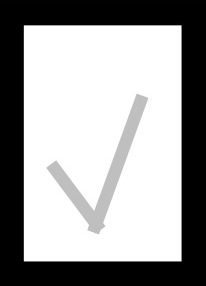
　　　　　　 None

**B. 【After the second dose】Have any physical symptoms appeared?**

**
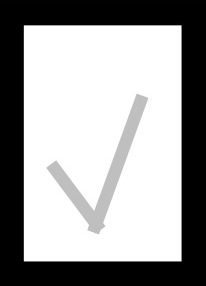

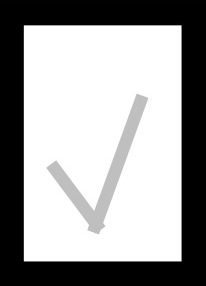
**　 No　　　 Yes

**B-1.** **If you answered "Yes", please mark the symptoms that apply to you.**

**
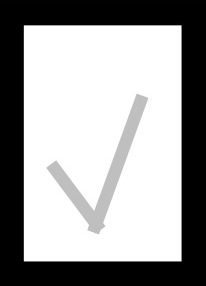

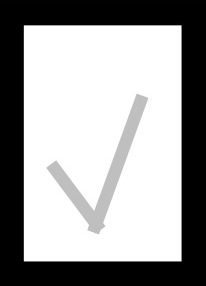
**
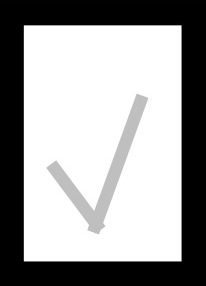
 Fever Headache GI symptoms（nausea, vomiting, diarrhea）


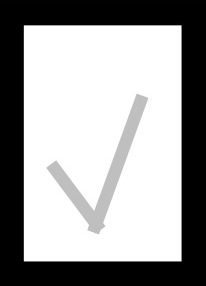

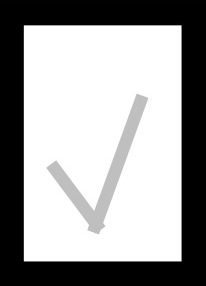
　Local reactions（pain, swelling, redness, itch）　Malaise


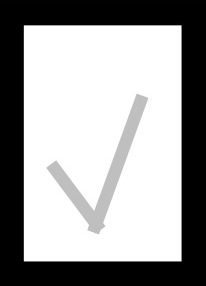
　　COVID arm


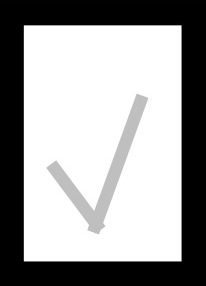
　Others（Details: 　　　　　　　　　　　　　　　　　　　　）

**B-2. To what extent have your physical symptoms affected your life?**

**
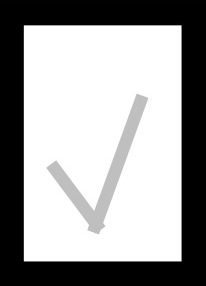
**　　　Major impact（ex. Couldn’t work due to AE）


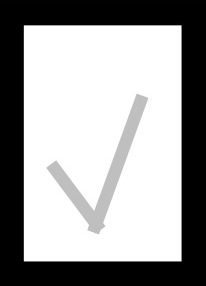
Moderate impact（ex. Work efficiency deteriorated.）


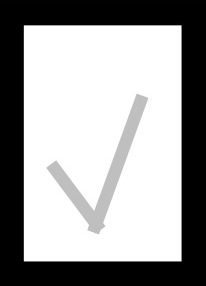
Little impact


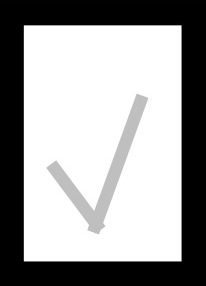
　　　　　　 None

**C. After the first or second vaccination, did you visit a hospital due to illness?**


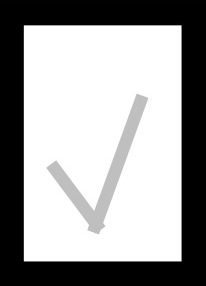
Hospitalization（Diagnosis：　　　　　　　　　　　　　　　）

Outpatient clinics（Diagnosis：　　　　　　　 　　　　　　）

No

**③History of COVID-19**

　・Have you ever had COVID-19?

　　　　 No　　 Yes

　・Date of onset^※^ (YYYY/MM/DD)：（ / / 　）

　・Severity： Asymptomatic　 Mild Desaturation On ventilation

　・Long COVID： Yes（Details：　 　　） No

※If asymptomatic, date of positive antigen or PCR test

**④We would like to ask you about your own thoughts and feelings about vaccines**

１．What were the factors that led you to decide on vaccination? (multiple selections)

　　　Everyone would get vaccinated

Not to be considered antivaxxer

Fear of getting an infection

Fear of serious illness

Fear of long COVID

Concern of getting others infected

To go out and eat out

To travel abroad (study abroad, business trip, etc.)

Others（Details :　　　　 　　　　　　　　　　　　　　　　　　　）

２．From which media do you obtain vaccine-related information? (multiple selections)

Televisions

Newspapers

Books

Websites（Details :　　　　　　　　　　　　　　　　　　　　　　）

SNS

Public institutions（Details：　 　　　　　　　　　　　　　　　　）

Medical papers

University information

Word of mouth

Others（Details :　　　　　　　　　　　　　　　　　　　　　　　）

３．**Pre-vaccination thoughts and feelings**

　　　Please circle the number that best applies.

**・Did you hesitate to get vaccinated?**

No

Yes

（If 1 or 2, please write the reason）

**・Did you expect the vaccine to work?**

No

Yes

**・Were you worried about short-term adverse reactions?**

No

Yes

**・Were you worried about long-term adverse reactions?**

No

Yes

**・Did you expect that mass vaccination would loosen behavioral restrictions?**

Yes

No

**・Were the people around you in favor of vaccination?**

No

Yes

４．Current thoughts and feelings

**・Do you think you were right to vaccinate?**

Yes

No

（If 1 or 2, please write the reason）

**・Are you worried about long-term adverse reactions?**

Yes

No

（If 1 or 2, please write the reason）

**・Are you planning to get a third vaccination?**

Yes

No

（If 1 or 2, please write the reason）

**・Do you believe in the efficacy of vaccines in general?**

Yes

No

**・Do you believe in the safty of vaccines in general?**

Yes

No

**・Would you like to receive a third vaccination even if you have to pay for it?**

No

Yes

→**How much do you pay for 3rd vaccination? (yen)**

≦1000 　2000　 3000　 4000　 5000

6000　 7000　 8000　 9000 ≧10000
